# Clinical utility of tissue Doppler imaging in patients with acute myocardial infarction complicated by cardiogenic shock

**DOI:** 10.1186/1476-7120-6-11

**Published:** 2008-03-20

**Authors:** Adnan K Hameed, Tirath Gosal, Tielan Fang, Roien Ahmadie, Matthew Lytwyn, Ivan Barac, Shelley Zieroth, Farrukh Hussain, Davinder S Jassal

**Affiliations:** 1 Department of Medicine, University of Manitoba, Winnipeg, Manitoba, Canada; 2 Cardiology Division, Department of Cardiac Sciences, St. Boniface General Hospital, University of Manitoba, Winnipeg, Manitoba, Canada; 3 Institute of Cardiovascular Sciences, Cardiology Division, Department of Cardiac Sciences, St. Boniface General Hospital, University of Manitoba, Winnipeg, Manitoba, Canada; 4 Department of Radiology, St. Boniface General Hospital, University of Manitoba, Winnipeg, Manitoba, Canada

## Abstract

**Background:**

Echocardiography is widely used in the management of patients with cardiogenic shock (CS). Left ventricular ejection fraction (EF) has been shown to be an independent predictor of survival in CS. Tissue Doppler Imaging (TDI) is a sensitive echocardiographic technique that allows for the early quantitative assessment of regional left ventricular dysfunction. TDI derived indices, including systolic velocity (S'), early (E') and late (A') diastolic velocities of the lateral mitral annulus, are reduced in heart failure patients (EF < 30%) and portend a poor prognosis. In CS patients, the application of TDI prior to revascularization remains unknown.

**Objective:**

To characterize TDI derived indices in CS patients as compared to patients with chronic CHF.

**Methods:**

Between 2006 and 2007, 100 patients were retrospectively evaluated who underwent echocardiography for assessment of LV systolic function. This population included: Group I) 50 patients (30 males, 57 ± 13 years) with chronic CHF as controls; and Group II) 50 patients (29 males, 58 ± 10 years) with CS. Spectral Doppler indices including peak early (E) and late (A) transmitral velocities, E/A ratio, and E-wave deceleration time were determined. Tissue Doppler indices including S', E' and A' velocities of the lateral annulus were measured.

**Results:**

Of the entire cohort, the mean LVEF was 25 ± 5%. Cardiogenic shock patients demonstrated significantly lower lateral S', E' and a higher E/E' ratio (p < 0.01), as compared to CHF patients. The in-hospital mortality in the CHF cohort was 5% as compared to the CS group with an in hospital mortality of 40%. In the subset of CS patients (n = 30) who survived, the mean S' at presentation was higher as compared to those patients who died in hospital (3.5 ± 0.5 vs. 1.8 ± 0.5 cm/s).

**Conclusion:**

Despite similar reduction in LV systolic function, CS patients have reduced myocardial velocities and higher filling pressures using TDI, as compared to CHF patients. Whether TDI could be a reliable tool to determine CS patients with the best chance of recovery following revascularization is yet to be determined.

## Background

Cardiogenic shock (CS) is a state of inadequate tissue perfusion due to cardiac dysfunction. It is a potentially lethal complication that occurs in 10% of all cases of acute myocardial infarction (MI) [1]. Between 1995 and 2004, the National Registry of Myocardial Infarction database recorded 300 000 ST elevation MI's of which 8.6% presented with CS [2]. In patients hospitalized for either myocardial infarction, decompensated heart failure or following cardiac surgery, CS was the leading cause of death resulting in mortality rates of up to 80% [2]. Although rapid stabilization and treatment of reversible causes via early revascularization is a priority, the mortality rate due to CS in the current era remains high [3,4].

Prognostic echocardiographic factors for determination of early recovery after CS are limited. Short and long term mortality appears to be associated with initial depressed LV systolic function and mitral regurgitation (MR) as assessed by echocardiography [5]. In patients with LV ejection fraction (EF) less than 30%, survival at one year was 24% versus 56% for those with preserved systolic function [5].

Tissue Doppler imaging (TDI) is a sensitive, noninvasive echocardiographic method that records velocity of tissue motion within the myocardium. TDI has been evaluated in both in vitro and in vivo studies, allowing for the quantitative assessment of both global and regional function of the myocardium [6]. Indices derived from TDI, including systolic velocity (S'), early (E') and late (A') diastolic velocities of the lateral mitral annulus, are reduced in heart failure patients (EF < 30%) and portend a poor prognosis. Transmitral to early diastolic velocity ratio (E/E') obtained via TDI correlates strongly with LV filling pressures [7]. An E/E' ratio > 10 identified a pulmonary capillary wedge pressure (PCWP) > 15 mm Hg with a sensitivity of 92% and a specificity of 80% [8]. In addition to chronic CHF, a higher E/E' has also been shown to correlate with a worse prognosis in acute myocardial ischemia and hypertension [9-12]. The application of TDI prior to revascularization in patients presenting with CS remains unknown.

The objective of this study was to describe TDI derived indices in patients presenting with cardiogenic shock after acute myocardial infarction, prior to percutaneous revascularization.

## Methods

### Patient Population

Between 2006 and 2007, 100 consecutive patients were retrospectively evaluated who underwent echocardiography for assessment of LV systolic function. This population included: Group I) 50 patients with chronic CHF as age and sex matched controls; and Group II) 50 patients with CS. The CHF group was defined as LVEF < 40%, NYHA class III or IV status, with documented severe three vessel coronary artery disease on cardiac catheterization, requiring hospitalization for decompensated heart failure.

All patients in the cardiogenic shock (CS) group presented with an acute myocardial infarction defined as a rise and/or fall of cardiac biomarkers with at least one value above the 99^th ^percentile of the upper reference limit. Additionally, all patients had to either have symptoms of ischemia, ECG changes of new ischemia (new ST-T changes or new left bundle branch block), or the development of pathological Q waves on EKG [13]. Cardiogenic shock was diagnosed if the patient satisfied all of the following clinical and hemodynamic criteria: i) hypotension (a systolic blood pressure < 90 mmHg for at least 30 minutes or the need for supportive measures to maintain a systolic blood pressure ≥ 90 mmHg); ii) a cardiac index ≤ 2.2 l/min per m^2^; iii) and a pulmonary capillary wedge pressure ≥ 15 mmHg in the setting of an acute ischemic insult. All CS patients met the above criteria. The medical records of all 100 patients were extensively reviewed for baseline demographic and hemodynamic data. The retrospective study was approved by the local institutional review board.

### Echocardiography

Parasternal and apical views were obtained using the GE Vivid 7 (GE Medical Systems, Milwaukee, WI) standard echocardiographic system and multifrequency transducer with tissue Doppler capability. Standard two dimensional images, M-mode, spectral and color Doppler, and TDI were performed. All echoes were performed within 6 hours of the patient's initial admission to hospital. For the patients in CS, all echo parameters were obtained prior to revascularization.

Left ventricular (LV) inter-ventricular septal thickness (IVS), posterior wall thickness (PWT), and LV ejection fraction (EF) were determined from 2-dimensional images according to established criteria [14,15].

Left ventricular diastolic function was assessed using both conventional and novel diastolic parameters. Transmitral LV filling velocities at the tips of the mitral valve leaflets were obtained from the apical 4-chamber view using pulsed wave Doppler echocardiography. The transmitral LV filling signal was traced manually and the following variables were obtained: peak early (E) and late (A) transmitral velocities, and E/A ratio.

Tissue-Doppler derived indices were recorded at the lateral mitral annulus. A sample volume of 6 × 6 mm was positioned along the basal lateral wall of the apical 4 chamber view. These indices included systolic velocities (S'), early diastolic (E') velocities and late diastolic (A') velocities. Finally, the dimensionless index of E/E' was calculated. The estimated left atrial pressure was subsequently calculated as E/E' × 1.25 + 1.9 [7].

### Statistics

The data are summarized as mean ± SD or number (percentage). Chi-square or Fisher exact tests were applied to compare categorical variables. A Student t-testing was used to compare parameters between both groups. A p value < 0.05 was considered significant. The Statistical Analysis System 8.01 (SAS Insitute, Cary, NC) was used to perform the analysis.

## Results

### Baseline Characteristics

The total population included 100 patients (mean age 57 ± 12, range 43 to 77). Group I consisted of 50 patients (30 males, 57 ± 13 years) with chronic CHF as controls. Group II consisted of 50 patients (29 males, 58 ± 10 years) with CS. Baseline characteristics are shown in Table 1. The two groups were similar with respect to baseline demographics, cardiovascular risk factors, and 2D echocardiographic parameters. Despite the difference in concomitant medication use amongst both groups (Table 1), with all patients in the CS group being on inotropic support, the HR, SBP and DBP were similar for assessment of conventional diastolic and TDI parameters (Table 2). Of the entire cohort, the mean LVEF was 25 ± 5%.

**Table 1:** Clinical and 2-D echocardiographic findings in all patients (n = 100)

**Characteristics**	**CHF (n = 50)**	**CS (n = 50)**	**p-value**
Age (y)	57 ± 13	58 ± 10	0.88
Male Gender (%)	30 (60)	29 (58)	1.00
Diabetes mellitus (%)	30 (60)	32 (64)	0.90
Hypertension (%)	35 (70)	37 (74)	0.78
Dyslipidemia (%)	25 (50)	23 (46)	0.90
Smoking history (%)	34 (68)	32 (64)	0.82

***Cardiac medications***			

Beta blockers (%)	50 (100)	10 (20)	< 0.05
ACE inhibitors (%)	48 (96)	5 (10)	< 0.05
Digoxin (%)	25 (50)	5 (10)	< 0.05
Spironolactone (%)	32 (64)	0 (0)	< 0.05
Inotropic support (%)	0 (0)	50 (100)	< 0.05

***Left Heart Dimensions***			

IVS (mm)	10 ± 2	11 ± 2	0.87
PWT (mm)	11 ± 2	11 ± 1	0.90
LVEDD (mm)	60 ± 2	59 ± 3	0.85
EF (%)	25 ± 6	23 ± 7	0.82

Values are mean ± SD (percentage). CHF, congestive heart failure; CS, cardiogenic shock; y, years; IVS, interventricular septal thickness; PWT, posterior wall thickness; LVEDD, left ventricular end diastolic diameter; EF, ejection fraction. p < 0.05* was considered significant.

**Table 2:** Hemodynamic data and Doppler echocardiographic findings in all patients (n = 100)

**Characteristics**	**CHF (n = 50)**	**CS (n = 50)**	**p-value**
***Hemodynamic Data***			

HR (bpm)	81 ± 11	84 ± 7	0.76
SBP (mm Hg)	87 ± 7	86 ± 10	0.72
DBP (mm Hg)	50 ± 4	48 ± 8	0.68

***Doppler echocardiography***			

Mitral E velocity (cm/s)	71 ± 10	72 ± 8	0.74
Mitral A velocity (cm/s)	64 ± 13	66 ± 12	0.72
E/A ratio	1.03 ± 0.3	1.04 ± 0.4	0.78

***Doppler tissue imaging***			

S' (cm/s)	6.2 ± 1.3	3.0 ± 0.9	< 0.01
E' (cm/s)	5.1 ± 1.1	3.2 ± 1.2	< 0.01
A' (cm/s)	3.8 ± 1.0	3.2 ± 1.1	0.92
E/E' (lateral)	13 ± 3	22 ± 3	< 0.01
LAP (mm Hg)	19 ± 4	31 ± 4	< 0.01

Values are mean ± SD (percentage). CHF, congestive heart failure; CS, cardiogenic shock; HR, heart rate; SBP, systolic blood pressure; DBP, diastolic blood pressure; LAP, estimated left atrial pressure; p < 0.05* was considered significant.

### Diastolic Echocardiographic Parameters

**Figure 1:**
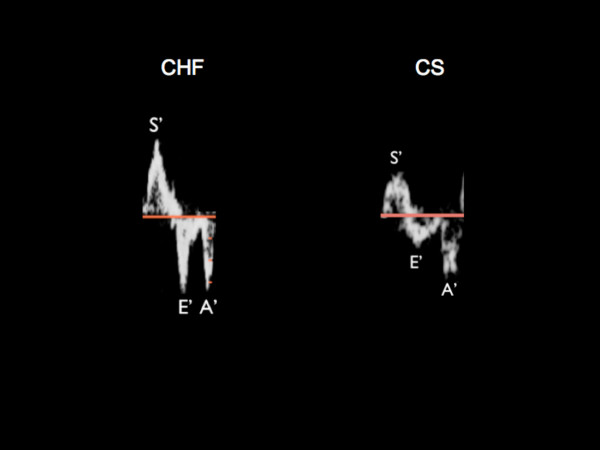
Representative TDI velocities of the lateral annulus including S', E' and A' in a patient with congestive heart failure (CHF) and in a patient with cardiogenic shock (CS) with similar LVEF.

Figure 1 illustrates examples of TDI annular parameters in a patient with CHF and a different patient with CS. All 100 patients had abnormal diastolic function, either having a pseudonormal pattern (n = 62) or a delayed relaxation pattern (n = 38) based on conventional diastolic parameters. There were no significant differences between the two groups with respect to heart rate, systolic blood pressure, and diastolic blood pressure (Table 2). All patients were in sinus rhythm. Individuals with CS demonstrated significantly lower lateral S' and E' (5.2 ± 1.3 cm/s vs. 2.1 ± 0.9 cm/s and 5.1 ± 1.1 cm/s vs. 3.4 ± 1.2 cm/s respectively) as compared to CHF patients. In addition, the E/E' ratio and estimated left atrial pressure (LAP) were elevated in CS patients as compared to CHF patients (Table 2).

### Clinical outcomes

**Figure 2:**
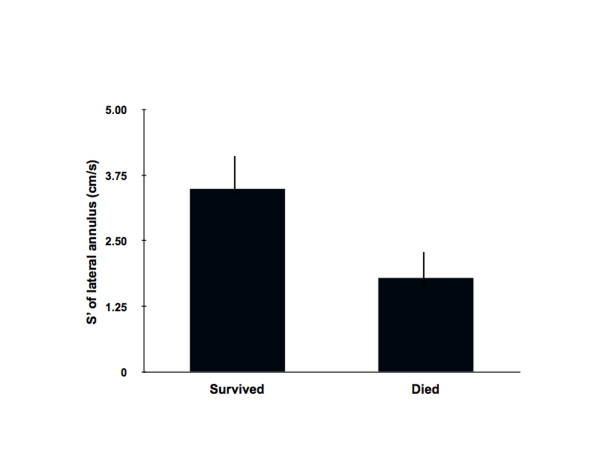
**In the CS group, 30 patients survived in-hospital and 20 patients died.** The mean S' at presentation, prior to revascularization, was higher in the survivors as compared to those patients who died (3.5 ± 0.5 vs. 1.8 ± 0.5 cm/s).

The mean duration of hospitalization for the CHF and CS patients were 10 ± 3 and 12 ± 4 days respectively. The in-hospital mortality in the CHF cohort was 5% as compared to the CS group with an in hospital mortality of 40%. In the subset of CS patients (n = 30) who survived in-hospital, the mean S' at presentation, prior to revascularization, was higher as compared to those patients who died (3.5 ± 0.5 vs. 1.8 ± 0.5 cm/s) as shown in Figure 2. At 6 months of followup, out of hospital mortality was 15% for CHF patients and 60% for CS patients.

## Discussion

Cardiogenic shock is the leading cause of death in patients with acute myocardial infarction with an in-hospital mortality rate greater than 50% [2]. A variety of structural and functional abnormalities are identifiable on echocardiography in patients presenting with acute CS [5]. Although Picard et al demonstrated reduced LV systolic function and mitral regurgitation as predictors of survival and response to early revascularization in CS [5], our study is the first to characterize a reduction in TDI derived indices in CS patients. Despite similar reductions in LV systolic function, our study demonstrated CS patients have reduced myocardial velocities and higher filling pressures using TDI as compared to CHF patients.

Mitral annular systolic velocity (S') reflects the long axis motion of the ventricle which is an important component of LV systolic function [16]. Peak myocardial systolic velocity averaged from six sites around the mitral annulus correlates well with LV ejection fraction. A S' value greater than 7.5 cm/s had a sensitivity of 79% and a specificity of 88% in predicting normal global LV function [17]. Sub-endocardial fibers make a substantial contribution to long axis function, and are susceptible to a variety of cardiac pathologies. Hypertension, coronary artery disease and CHF have been shown to negatively affect these fibers and reduce S'. A reduction in S' correlates with increased morbidity and mortality in each of these disease states [11,18,19].

In our study, CS patients had a significantly lower S' compared to CHF patients. Shock has severe metabolic consequences, involving mainly energy metabolism, substrate utilization, and acid-base regulation. As longitudinally arranged subendocardial fibers are most vulnerable to ischemia and metabolic abnormalities, it is not an unexpected finding that LV base-apex contraction was abnormal in our CS population. Given the prognostic value in other cardiac disease states, a reduction of S' in CS may be of similar clinical importance in this patient population. In our study, albeit small numbers, the subset of patients in CS who survived demonstrated increased S' values on initial presentation, prior to revasculariation, as compared to those who died in-hospital.

Similar to systolic annular velocity (S'), early diastolic mitral annular (E') appears to be a strong independent predictor for the short and long-term prognosis of patients with cardiovascular disease. E' has been proposed as a useful index for the non-invasive evaluation of LV relaxation, that is relatively preload independent [20]. Recent studies have shown that a reduced E' predicts increased cardiovascular morbidity and mortality [11,14,19]. Wang et al demonstrated that an E' less than 3 cm/s was the best prognostic marker for long-term follow up in a population of patients with chronic hypertension [11].

In the current study, E' was significantly reduced in the CS population as compared to chronic CHF patients. The potential mechanism for this abnormality may relate to the acute increase in LV wall stress due to the ischemic insult in the CS state. The reduced E' may be a sensitive marker of regional relaxation abnormality reflected in the long axis dimension that could potentially be evident earlier than clinical manifestations of global left ventricular relaxation abnormality.

Since E' is reduced and diastolic mitral annular increases with higher filling pressures, the E/E' ratio correlates well with invasive pulmonary capillary wedge pressure (PCWP) measurements [8,20,21]. Elevated PCWP is associated with a higher mortality rate after acute MI and has been shown to carry independent prognostic information in CHF patients [22-24]. In our study, CS patients had an elevated E/E' ratio and LAP confirming raised LV filling pressures. The spectrum of diastolic abnormalities which may account for the elevated ratios include increased myocardial stiffness, reduced LV compliance, and elevated LV end-diastolic parameters. A restricted filling pattern tends to correlate with higher mortality at 30 days and 1 year in CS [22], and is known to be associated with a poor prognosis in CHF patients [23]. Hillis et al. previously established that in the setting of acute MI, an E/E' ratio greater than 15 can identify patients at an increased risk of mortality with a sensitivity of 70% and a specificity of 91% [24,25]. In addition, the prognostic value of E/E' was incremental to clinical factors and conventional echocardiographic parameters of LV systolic and diastolic function [24,25]. An elevated E/E' ratio in CS patients may be of comparable value in distinguishing this select population at higher risk for poor cardiovascular outcomes [26].

## Limitations

Similar to other studies using TDI, this methodology is affected by the quality of 2D images and cardiac translation, rotation, or both. Only lateral annular velocities were analyzed as septal annular velocities may be subjected to the influence of the right ventricle. Only the lateral wall of the left ventricle was routinely sampled with TDI as per routine in our echocardiography laboratory. However, lateral velocities may be affected by both translational effects and beam angle [20,27]. The reduced LV systolic function in the CS population may have represented myocardial stunning, and thus the true LVEF may have been underestimated in this subgroup. Tissue Doppler imaging parameters was not obtained immediately following revascularization nor at 6 month followup in the patient population. Lastly, this study is limited by the relatively small sample size and retrospective design. A larger, prospective study is needed in order to make more substantive conclusions regarding the clinical utility of tissue Doppler indices prior to percutaneous revascularization in patients with cardiogenic shock.

## Conclusion

Despite similar reduction in LV systolic function, CS patients have reduced myocardial velocities and higher filling pressures compared to chronic CHF patients. Whether TDI could be a reliable tool to determine CS patients with the best chance of recovery following revascularization is yet to be determined.

## References

[B1] Babaey A, Frederick PD, Pasta DJ, Every N, Sichrovsky T, Hochman JS, NRMI Investigators Trends in management and outcomes of patients with acute myocardial infarction complicated by cardiogenic shock. JAMA.

[B2] Hochman JS, Boland J, Sleeper LA, Porway M, Brinker J, Col J, Jacobs A, Slater J, Miller D, Wasserman H, Menegus M, Talley D (1995). Current spectrum of cardiogenic shock and effect of early revascularization on mortality. Results of an International Registry. Circulation.

[B3] Hochman JS, Sleeper LA, Webb JG, Sanborn TA, White HD, Talley JD, Buller CE, Jacobs AK, Slater JN, McKinlay SM, Picard MH, Menegus MA, Boland J, Dzavik V, Thompson CR, Wong SC, Steingart R, Forman R, Aylward P, Godfrey E, Nickens PD (1999). Early revascularization in acute myocardial infarction complicated by cardiogenic shock. N Engl J Med.

[B4] Hochman JS, Sleeper LA, White HD, Dzavik W, Wong SC, Menon V, Webb J, Steingart R, Picard M, Menegus M, Boland J, Sanborn T, Buller C, Modur S, Forman R, Nickens PD, Jacobs AK, Slater J, LeJemtel T (2001). One-year survival following early revascularization for cardiogenic shock. JAMA.

[B5] Picard MH, Davindoff R, Sleeper LA, Mendes LA, Thompson CR, Dzavik V, Steingart R, Gin K, White HD, Hochman JS (2003). Echocardiographic predictors of survival and response to early revascularization in cardiogenic shock. Circulation.

[B6] Miyatake K, Yamagishi M, Tanaka N, Uematsu M, Yamazaki N, Mine Y, Sano A, Hirama M (1995). New method for evolution of left ventricular wall motion by color-coded tissue Doppler imaging. In vitro and in vivo studies. J Am Coll Cardio.

[B7] Nagueh SF, Middleton KJ, Kopelen HA, Zoghbi WA, Quinones MA (1997). Doppler tissue imaging: a non-invasive technique for evaluation of left ventricular relaxation and estimation of filling pressures. J Am Coll Cardiol.

[B8] Gulati VK, Katz WE, Follansbee WP, Gorcsan J (1996). Mitral annular descent velocity by tissue Doppler echocardiography as an Index of global left ventricular function. Am J Cardiol.

[B9] Dokainish H, Zoghbi WA, Lakkis NM, Ambriz E, Patel R, Quinones MA, Nagueh SF (2005). Incremental predictive power of B-type natriuretic peptide and tissue Doppler echocardiography in prognosis of patient with congestive heart failure. J Am Coll Cardiol.

[B10] Yu C, Sanderson JE, Marwick TH, Oh JK (2007). Tissue Doppler imaging: a new prognosticator in cardiovascular diseases. J Am Coll Cardiol.

[B11] Wang m, Yip GW, Wang AY, Zhang Y, Ho PY, Ise MK, Yu CM, Sanderson JE (2005). Tissue Doppler imaging provides incremental prognostic value in patients with systemic hypertension and left ventricular hypertrophy. J Hypertens.

[B12] Arques S, Roux E, Luccioni R Current clinical applications of spectral tissue Doppler echocardiography (E/E' ratio) as a noninvasive surrogate for left ventricular diastolic pressures in the diagnosis of heart failure with preserved left ventricular systolic function. Cardiovasc Ultrasound.

[B13] Thygesen K, Alpert JS, White HD, on behalf of the joint ESC/ACCF/AHA/WHF (2007). Task force for the Redefinition of Myocardial Infarction. Circulation.

[B14] Lang RB, Bierig M, Devereaux RB (2005). Chamber quantification writing group: American Society of Echocardiography's Guidelines and Standards Committee, European Association of Echocardiography. Recommendations for chamber quantification: A report from the American Society of Echocardiography's Guidelines and Standards Committee and the Chamber Writing Group, developed in conjunction with the European Association of Echocardiography, a branch of the European Society of Cardiology. J Am Soc Echocardiogr.

[B15] Devereux RB, Alonso DR, Lutas EM, Gottlieb GJ, Campo E, Sachs I, Reichek N (1986). Echocardiographic assessment of left ventricular hypertrophy: comparison to necropsy findings. Am J Cardiol.

[B16] Henein MY, Gibson DG (1999). Long axis function in disease. Heart.

[B17] Alam M, Wardell J, Andersson E, Samad BA, Nordlander R (2000). Effects of first myocardial infarction on left ventricular systolic and diastolic function with the use of mitral annular velocity determined by pulsed wave Doppler tissue Imaging. J Am Soc Echocardiogr.

[B18] Marwick TH, Case C, Leano R, Short L, Baglin T, Cain P, Garrahy P (2004). Use of tissue Doppler imaging to facilitate the prediction of events in patients with abnormal left ventricular function by dobutamine echocardiography. Am J Cardiol.

[B19] Nikitin NP, Loh PH, Silva R, Ghosh J, Khaleva OY, Goode K, Rigby AS, Alamgir F, Clark AL, Cleland JG (2006). Prognostic value of systolic mitral annular velocity measured with Doppler tissue imaging in patients with chronic heart failure caused by left ventricular systolic dysfunction. Heart.

[B20] Sohn DW, Chai IH, Lee DJ, Kim HC, Kim HS, Oh BH, Lee MM, Park YB, Choi YS, Seo JD, Lee YW (1997). Assessment of mitral annulus velocity by Doppler tissue imaging in the evaluation of left ventricular diastolic function. J Am Coll Cardiol.

[B21] Nagueh SF, Sun H, Kopelen HA, Middleton KJ, Khoury DS (2001). Hemodynamic determinants of mitral annulus diastolic velocities by tissue Doppler. J Am Coll Cardiol.

[B22] Reynolds HR, Anand SK, Fox JM, Harkness S, Dzavik V, White HD, Webb JG, Gin K, Hochman JS, Picard MH (2006). Restrictive physiology in cardiogenic shock: Observations from echocardiography. Am Heart J.

[B23] Xie GY, Berk MR, Smith MD, Gurley JC, DeMaria AN (1994). Prognostic value of Doppler transmitral flow patterns in patients with congestive heart failure. Jam Coll Cardiol.

[B24] Hillis GS, Moller JE, Pellikka PA, Gersh BJ, Wright RS, Ommen SR, Reeder GS, Oh JK (2004). Non-invasive estimation of left ventricular filling pressure by E/E' is a powerful predictor of survival after acute myocardial infarction. J Am Coll Cardiol.

[B25] Hillis GS, Ujino K, Mulvagh SL, Hagen ME, Oh JK (2006). Echocardiographic indices of increased left ventricular filling pressure and dilation after acute myocardial infarction. J Am Soc Echocardiogr.

[B26] Vermes E, Houël R, Simon M, Le Besnerais P, Loisance D (2000). Tissue Doppler imaging to predict myocardial recovery during mechanical circulatory support. Ann Thorac Surg.

[B27] Dokainish H, Zoghbi WA, Lakkis NM, Ambriz E, Patel R, Quinones MA, Nagueh SF (2005). Incremental predictive power of B-type natriuretic peptide and tissue Doppler echocardiography in the prognosis of patients with congestive heart failure. J Am Coll Cardiol.

